# Chance mechanisms affecting the burden of metastases

**DOI:** 10.1186/1471-2407-5-138

**Published:** 2005-10-26

**Authors:** Wayne S Kendal

**Affiliations:** 1 Division of Radiation Oncology, The Ottawa Hospital Regional Cancer Centre, 503 Smyth, Ottawa, Ontario, K1H 1C4 Canada and The Ottawa Hospital Research Institute, Ottawa, Ontario, K1H 8L6 Canada

## Abstract

**Background:**

The burden of cancer metastases within an individual is commonly used to clinically characterize a tumor's biological behavior. Assessments like these implicitly assume that spurious effects can be discounted. Here the influence of chance on the burden of metastasis is studied to determine whether or not this assumption is valid.

**Methods:**

Monte Carlo simulations were performed to estimate tumor burdens sustained by individuals with cancer, based upon empirically derived and validated models for the number and size distributions of metastases. Factors related to the intrinsic metastatic potential of tumors and their host microenvironments were kept constant, to more clearly demonstrate the contribution from chance.

**Results:**

Under otherwise identical conditions, both the simulated numbers and the sizes of metastases were highly variable. Comparable individuals could sustain anywhere from no metastases to scores of metastases, and the sizes of the metastases ranged from microscopic to macroscopic. Despite the marked variability in the number and sizes of the metastases, their respective growth times were rather more narrowly distributed. In such situations multiple occult metastases could develop into fully overt lesions within a comparatively short time period.

**Conclusion:**

Chance can have a major effect on the burden of metastases. Random variability can be so great as to make individual assessments of tumor biology unreliable, yet constrained enough to lead to the apparently simultaneous appearance of multiple overt metastases.

## Background

The burden of metastatic disease can be an important determinant in cancer management [1]. With many of the common epithelial cancers, successful resection of the primary tumor may be all that is required for cure, provided that there are no metastases. If the numbers of metastases are limited, it may be possible to resect them along with the primary tumor for curative intent [2-4]. Moreover, adjuvant therapy may cure individuals who might otherwise succumb, provided that the metastatic burden is small and their disease is responsive to therapy [5,6].

In those individuals who have had their metastatic disease resected, the number of metastases removed tends to have an adverse correlation with survival [3,7-10]. In addition, the total volume of resected metastases may provide an even stronger prognostic indicator [11]. Clinicians have often considered the number of metastases sustained by an individual to be a biological measure of intrinsic tumor behavior [12-16]. In keeping with this view, both clinicians [17] and experimentalists [18-22] have come to equate the metastatic potential of tumors with the numbers of organ metastases.

There is, however, a critical distinction between human clinical observations and animal experiments: Animal experiments can be repeated under more or less uniform conditions; human observations are usually isolated and more heterogeneous. Even so, controlled animal experiments tend to exhibit considerable heterogeneity in the numbers of hematogenous metastases [18,21,23]. In some cases the observed experimental heterogeneity can be attributable to pre-existent variant cells within tumor cell populations [18,24], but even within groups of syngeneic animals treated with the same tumor cells, the numbers of resultant metastases can be quite variable [25,26]. The metastatic heterogeneity, evident with these animal studies, is also evident with human studies [27]. This pattern can be attributed to physiologic heterogeneities in regional organ blood flow and, combined with counting (Poisson) statistics, the two processes can be used to explain the disparities in the numbers of haematogenous metastases evident to individuals under otherwise similar conditions [28,29].

Human autopsy studies have also revealed that the sizes of metastases within individuals can be quite variable [17,30]. In individuals with multiple metastases the frequency distribution for the sizes of their metastases approximates a lognormal form, which presumably relates to a normal distribution for the growth times of metastases, provided that the growth of the secondary deposits is approximately exponential. These normally distributed growth times can, in turn, be explained by the summation of the times required for the multiple sequential steps deemed necessary for tumor growth and metastasis [31].

On this basis, both the number of metastases and the sizes of metastases can manifest random influences, in addition to being affected by the tumor's metastatic potential and the host microenvironment. Since both the numbers of metastases and the total tumor burden have been represented as measures of tumor biology [11-16], it would be important to determine whether or not these additional spurious influences might obscure the biological assessments of malignant behavior.

The central point to be illustrated here is that biologically similar tumors may give rise to highly variable numbers or volumes of metastases. Because of this, the clinically apparent metastatic burden may not provide a good measure of the biological characteristics of a tumor. Animal experiments have shown that the metastatic burden does indeed reflect the biological properties of a tumor, but spurious influences are commonly evident within such experiments [18,21,23]. For this reason animal experiments must be repeated a number of times in order to ascertain the biological properties and to account for chance variations. Clinicians are not able to do this when assessing the biological potential of an individual's cancer. Clinical decisions may be made on the basis of the evident burden of disease, without knowledge as to the degree that chance might have influenced the clinical presentation. For this reason it would be important to demonstrate the potential influence that chance might have on the metastatic burden.

To this end models for the number distribution [29] and size distribution [31] of hematogenous metastases will be utilized here to simulate the metastatic burden within a hypothetical series of identical cancer patients. Since it was not possible to control all the biological and temporal variables in actual human clinical series, simulation was chosen as the most practical means to assess the heterogeneity in metastatic burden that could be attributable to chance.

## Methods

Monte Carlo simulations were performed for the numbers of hematogenous metastases within individuals where the intrinsic biological conditions of the tumor and the host microenvironment were presumed to remain constant by maintaining the same mean and variance for the number of metastases in each simulation. Granted, this approach represents a simplification of the influence of tumor growth factors, and their receptors, the production of angiogenic factors, the capacity for motility and invasion, or the capacity for aggregation and deformability which all may affect a tumor's intrinsic capacity to metastasise and proliferate [32]. The interplay of pararcrine and endocrine growth factors, neovascularization, platelet aggregation and the action of immune cells and their products are additional factors that contribute to the host microenvironment [32] were similarly treated. The precise way that these factors might exert their influences goes beyond the aim of this manuscript, and a phenomenological approach was taken instead. To this end the Poisson negative binomial (PNB) distribution can accurately represent the variations in the numbers of hematogenous metastases sustained by individuals under otherwise identical conditions [29], and it was thus used to represent the frequency distribution for the numbers of metastases (see the Additional file 1 for details regarding the PNB distribution). In this model, variations in the numbers of metastases can be attributed to both random chance and to heterogeneity in regional organ blood flow [28,33].

The simulation parameters were chosen to reflect a mean number of about 8 metastases, and a variance of 57 metastases^2^. This was within the range of observation of controlled metastasis experiments of the murine B16 F10 melanoma in lung [29], and well within the range of the much more heterogeneous clinical data from humans draw from lung and liver metastases secondary to sarcomas, lung carcinomas, and various gastrointestinal carcinomas [27]. This choice of parameters also afforded a sizeable fraction of cases with no metastases and was made with the view that the mean number of resultant metastases was in itself not critical, since the aim of the simulation was to illustrate the variability in the numbers of metastases that might occur, under otherwise constant intrinsic biological conditions.

Admittedly the choice of parameters here was arbitrary, and designed to illustrate (not to prove) the potential variability in the numbers of metastases. In simulations such as these, the end results are predetermined by the choice of models and parameters. However, in order to provide realistic results, the models and parameters used were chosen to emulate laboratory and clinicopathological observation as closely as possible. Because the human data reflected populational heterogeneity, with respect to growth rates of primary tumors and their metastases and the rates of metastatic dissemination, as well as differences in times of the onset of the primary tumor relative to the assessment of the numbers of metastases of each individual's disease, these human data could serve only as an outside bounds for the potential ranges of these parameters. Controlled experiments from the murine B16 F10 melanoma were extrapolated upon to provide somewhat closer bounds for the parameter choices.

A second set of Monte Carlo simulations was performed to yield the size distribution of metastases expected within individuals. Each metastasis was assumed to grow in accordance with a stochastic pure birth process [34] (see the Additional file 1). The growth times for the metastases within any individual were further assumed to be normally distributed, in accordance with deductions from previous human autopsy studies [31]. According to this model, the size variations of the metastases could be essentially attributed to random chance.

The growth times for the metastases, as defined from the establishment of each nascent (and growing) metastasis to the surgical removal of the primary tumor, was arbitrarily assumed to have a mean and standard deviation (SD) of 16 ± 1 volume doublings. After resection of the primary tumor, the metastases were assumed to grow for an additional 12 doublings before they were counted and sized. This sequence was chosen to emulate the clinical sequence of resection of a primary tumor, performed in an individual with no clinically detectable metastases, followed by restaging after a set time interval. As well, and as a first approximation, the growth rates of the metastases were assumed to be the same as that of the primary tumor.

These two simulations were combined into a third simulation, designed to provide the number and sizes of metastases that could be anticipated within a group of similar individuals. Fifty simulations were performed where the biological properties intrinsic to the primary tumor and the host microenvironment were assumed constant.

A further calculation was performed, based upon the size distribution of 4000 simulated metastases, in order to determine the proportion of clinically detectable metastases at various time intervals from the surgical resection of the primary tumor. The growth parameters were the same as in the previous calculations, and the clinical detection threshold was arbitrarily set at 10^9 ^cells (~1 cm^3^).

## Results

**Figure 1:**
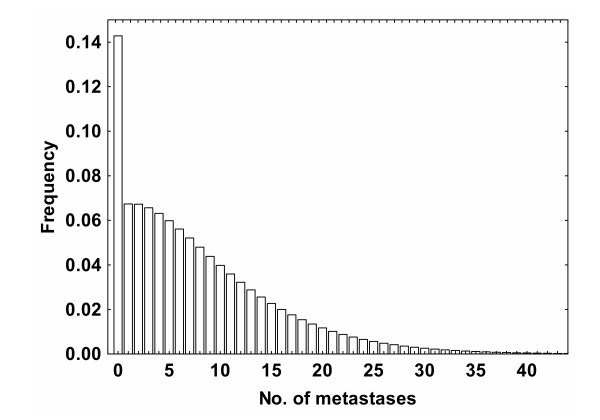
**Frequency distribution for the numbers of metastases**. Frequency distributions for the number of hematogenous metastases can be predicted based upon random counting statistics, modulated by regional organ blood flow heterogeneity. In this model the biological properties of the tumor cells and the host environment were otherwise assumed constant. The simulation was executed with a mean of 8 ± 7.6 (SD) metastases, in keeping with observations compiled from murine and human tumors.

Figure 1 provides the results of the simulation for the numbers of hematogenous metastases. About 14% of the simulated cases sustained no metastases; the majority had 6 or fewer metastases, whereas a small minority exhibited as many as 40 metastases. The numbers of metastases sustained by individuals under identical conditions were thus quite variable, but they were consistent with the available clinical and pathological studies [27].

**Figure 2:**
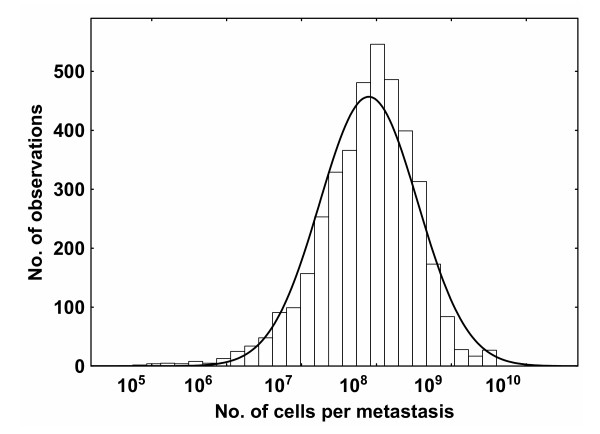
**Size distribution for metastases**. A model for the growth of metastases was based upon a stochastic birth process where the growth times of the individual colonies were normally distributed. Four thousand simulations were performed. The resultant frequency histogram for the logarithm of the number of cells per metastasis is shown here, and it approximated a normal distribution (solid line). This was equivalent to a lognormal distribution for the sizes of metastases.

Simulations were also performed for the size distribution of metastases in this hypothetical series of patients. A total of 4,000 simulations yielded a mean metastasis size of 4 × 10^8 ^cells (range 2 × 10^4 ^to 8 × 10^9 ^cells; SD 8 × 10^8 ^cells). Figure 2 provides the frequency histogram for the logarithm of the number of cells per metastasis, as determined from the 4,000 simulations. The histogram approximated a normal distribution, and was consistent with the available data from human autopsy studies [17,31].

The final simulations were designed to emulate a hypothetical case series of 50 cancer patients. As before, the intrinsic biological properties of host and tumor were assumed identical, the primary tumors were removed at comparable times, and the resultant metastases were enumerated 12 volume-doubling times later.

**Figure 3:**
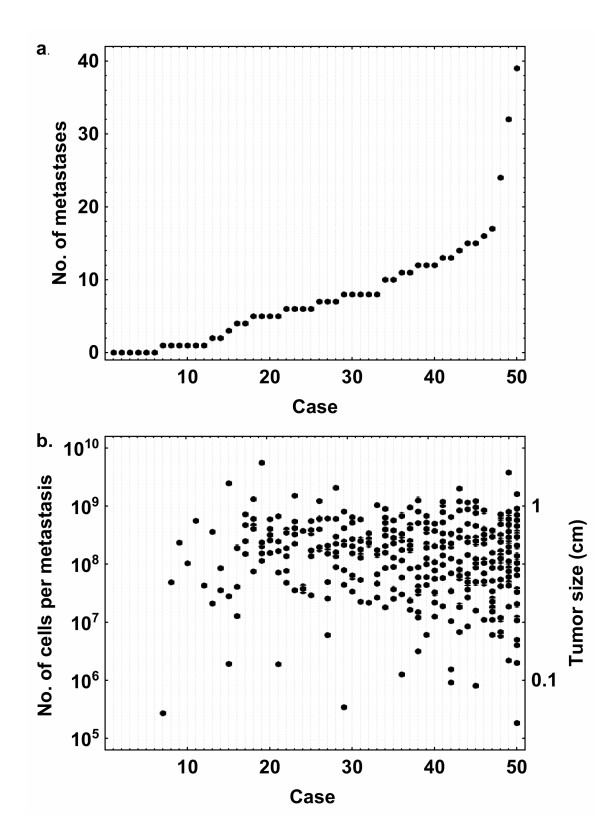
**Fifty simulations for the numbers and sizes of metastases**. In these simulations the primary tumors were presumed successfully resected from each host. All biological properties of the tumors and hosts were assumed constant. The only sources for deviation were random chance and heterogeneities in regional organ blood flow. In each simulation there were no clinically detectable metastases at the time of surgery, and the individuals were restaged after a time to allow for 12 volume doublings. **a. Simulated numbers of metastases**. Using the same parameterization as employed in Fig. 1, six cases sustained no metastases whereas the remaining cases sustained anywhere from one to just under forty metastases. **b. Simulated sizes of metastases**. The resultant sizes of metastases from each case are plotted here semi-logarithmically. The right axis provides the numbers of cells per metastasis; the left gives the corresponding diameters of the metastases. Many of these metastases would likely be below the threshold of conventional clinical detection (arbitrarily defined here as at 10^9 ^cells). Given additional time for growth the subclinical metastases would have also become overt.

Figure 3 provides the results from this simulated case series: Fig. 3a gives the numbers of simulated metastases. Six cases sustained no metastases; the majority of cases sustained less than 10 metastases; three cases sustained more than 20 metastases. Figure 3b provides the respective sizes of the metastases from each case. Most cases had metastases that consisted of fewer than 10^9 ^cells. Both the numbers and sizes of the metastases were highly variable, despite the identical biological conditions prerequisite to the simulations.

From Fig. 3b it can be seen that the majority of the simulated metastases would have been so small as to be effectively occult, given a detection threshold of 10^9 ^cells. However, only a small number of additional volume doublings would have been required for many of these occult metastases to become overt.

**Figure 4:**
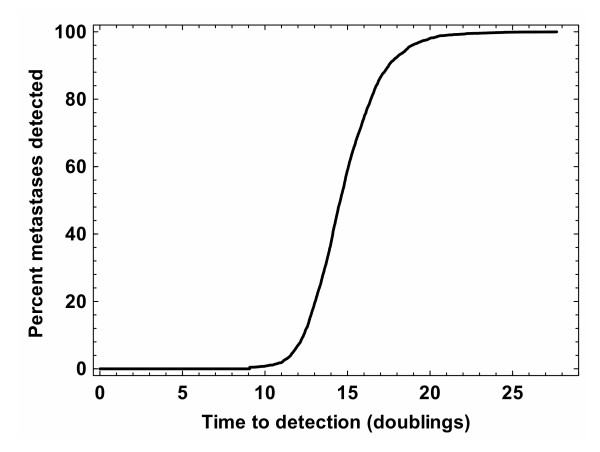
**Detection of metastases**. The percentage of clinically detectable metastases relative to the total number of metastases within a patient is plotted here versus the time interval from the removal of the primary tumor to detection. This plot was based upon the 4000 simulations cells. A latent period provided in Fig. 2, and the detection threshold was set at 10^9 ^was apparent, where the metastases remain clinically occult, followed by a period where the proportion of overt metastases rapidly increased.

To better demonstrate the kinetics of the transition from occult to overt metastases, the proportion of 4,000 simulated metastases that had reached a detection threshold of 10^9 ^cells was plotted versus the time interval from removal of the primary tumor. Figure 4 illustrates this graph. After a latent period of about a dozen doubling times, the first metastases would have appeared. With about an additional 3 volume doubling times beyond this, a majority of the remaining metastases would then have become detectable, as had been surmised from Fig. 3b.

The appearance of multiple overt metastases within a relatively short time period could be directly related to the growth time distribution for multiple metastases. As previously noted, this distribution can be approximated by the normal distribution [31]. The narrower this distribution is, the more likely that multiple metastases would seem to appear within a short time period.

## Discussion

How well do these simulations represent reality? The answer to this question depends, in part, upon the validity of the two models the simulations were based upon, one for the frequency distribution of the number of hematogenous metastases [29], the other for the size distribution of metastases [31]. The frequency distribution of metastases was derived from observations of thousands of humans and animals afflicted with cancer [25-27,29], and it was mechanistically explained in terms of the influence of physiological variations in regional organ blood on counting statistics [28,29,33]. The size distribution for hematogenous metastases was derived from detailed individual human autopsy studies of thousands of metastases, which agreed well with the lognormal distribution [17,30,31]. It was mechanistically based on the hypothesis of normally distributed growth times for exponentially growing metastases [31]. Both models seemed well grounded on observation, and they both had plausible mechanisms.

As a further requirement for the simulations to be realistic, the underlying models needed to yield results that were within the range of experience. Human data were not available for the numbers of hematogenous metastases sustained by syngeneic hosts, injected with cells from the same tumor and assessed at the same time in the natural history of the disease; but murine data were [18,21,26]. The simulations for the numbers of metastases were thus parameterised so as to fit within the observed range of the murine B16 F10 melanoma data. A mean number of 8 ± 8 (SD) metastases was chosen, and with a variance to mean ratio of *σ*^2^/*μ*~8, results which were similar to observations from clones of the B16 F10 murine melanoma in experimental metastasis assays with age matched syngenic mice [25]. These simulated results also fell within the bounds of the more heterogeneous clinical and pathological observations from humans [27].

With regards to the parameterisation for the size distribution of metastases, the simulated volumes (mean ± SD, 0.4 ± 0.8 cm^3^), and their coefficient of variation (*σ*/*μ*~2), fitted into the range observed from individual human autopsy studies [17,30]. The predictions from both of these Monte Carlo simulations were thus consistent with laboratory and human data.

What insights did these simulations provide into metastasis? The conventional model for hematogenous metastasis involves multiple sequential steps – the primary tumor must produce cells capable of metastasis, these cells must intravasate, exfoliate, successfully traverse the circulation system, arrest in a target organ favourable to growth, extravasate, form micrometastases, induce angiogenesis, and then proliferate [35,36]. At points along this pathway, tumor cells may also die or become dormant. The eventual burden of metastases presumably reflects the kinetic balances inherent to each step, as well as the relative time periods spent in dormancy and proliferation. The biological properties of the tumor cells, the host environment, and the biophysical aspects of the transport processes involved likely would affect metastasis, as would random events [37].

Much has been said and written about metastasis being a nonrandom process [38-43]. This dictum seems most appropriate when restricted to the organ predilections contingent to the seed and soil hypothesis [32,44], and to the anatomic pathways of regional metastasis [45,46]. However, the influence of random chance in metastasis has not as thoroughly been deliberated. To this end, the simulations preformed here showed that both the numbers and sizes of metastases within an individual could be affected in a major way by chance mechanisms.

At the same time the spurious underlying nature of metastasis has been well apparent to experimentalists. In the conventional experimental metastasis assay, and despite efforts to keep conditions constant, when syngeneic age-matched animals are injected with equal sized aliquots of the same tumor cell suspension, the resultant numbers of lung metastases can be quite variable [18-22]. Biologists have come to rely upon statistical tests to compare the metastatic potentials of different tumor variants; the median or mean number of metastases per group would then provide an index for the metastatic potential of the tumor, and the random variability could be quantitated by the variance of the number of metastases per each group [25,26]. This variability can be significant and it would be reasonable to postulate a similar degree of variability to be associated with individual human cases.

The real issue is that the burden of metastasis can be attributed partly to the biological properties of the tumor and partly to chance events. To understand how to use the observed metastatic burden in clinical practice, one must understand the relative importance of the deterministic and random components. Clinicians cannot rely upon multiple replicate observations, and their measured variance, to assess the random component as can be done in the animal laboratory. We are left to extrapolate from the laboratory experiments where the numbers of metastases in otherwise identical circumstances can vary considerably. The parameters of the simulations performed here were chosen to emulate human and animal observations as closely as possible. One obviously cannot prove that there exists a large random component to the metastatic burden by these means; however, one can provide a plausible example for the potential degree of variability that might be encountered in clinical situations.

Chance also seems to have a role in the relatively sudden appearance of multiple metastases, in persons who have previously been followed without apparent disease. Clinical experience indicates that it is not unusual for multiple metastases to seem to appear within a relative short time interval [47-52]. From the simulations presented here, such apparently synchronous emergence of multiple metastases within an individual could be attributable to a relatively compact distribution for their growth times. Admittedly there are also cases where multiple asynchronous metastases can manifest over a longer time course; in these cases presumably this distribution would not be so compact.

The aim of this study was to examine the variability in the metastatic burden, under circumstances where the metastatic potential of a tumor and the conditions within the host for tumor metastasis and growth could be considered constant. Earlier in this article, the significant variability in the numbers of experimental and spontaneous metastases amongst similarly treated animals was alluded to [18,21,26,53]. Because of this variability, laboratory scientists have come to rely upon multiple measurements from identically treated animals in order to assess the metastatic potential of tumors. Indeed, the variability can be such that animals from a treatment group might sustain multiple metastases while others from the same group remained metastasis-free. Similar disparities, if observed in human clinical series, might be construed as to represent the differing biological behavior of individual tumors [12-16]. However in the light of these experimental data, and the simulations provided here, these variations could reasonably be attributed to spurious events, rather than to inherent biological differences. For this reason it would appear to be difficult to draw inferences regarding the biological behavior of a tumor, based upon individual assessments of tumor burden.

## Conclusion

The burden of metastases sustained by an individual can presumably be influenced by the intrinsic biology of the tumor, the host microenvironment, the time available for tumor growth, and by the interaction of chance events with these processes. The origins of these chance events are not entirely clear; this could include both physical and biological processes. Nevertheless, such events could lead to a wide variation in number and sizes of metastases, under otherwise controlled conditions. The growth times of individual metastases are also affected by chance. But here the variability is more constrained, and consequently multiple occult metastases within an individual may relatively suddenly become clinically overt.

These particular kinetic properties of metastases, although influenced by tumor and host biology, can thus also be affected by chance, to a degree sufficient to potentially obscure the underlying biology. Clinicians will commonly make an assessment of the nature of a particular individual's cancer based upon the apparent burden of metastases. This simulation demonstrates that stochastic elements could potentially have a major influence on the burden of metastasis, to the point that it would seem unreliable, in any individual, to base assessments as to the biological potential of the cancer on the metastatic burden.

## List of abbreviations

PNB, Poisson negative binomial; PGF, probability generating function; CDF, cumulative distribution function; SD, standard deviation.

## Competing interests

The author(s) declare that they have no competing interests.

## Pre-publication history

The pre-publication history for this paper can be accessed here:



## Supplementary Material

Additional File 1 The distribution of the numbers of metastases. Click here for file
