# Supplementary material for: Chance mechanisms affecting the burden of metastases
Source: BMC Cancer. 2005 Oct 26;5:138. doi: 10.1186/1471-2407-5-138 (PMC1289278; doi:10.1186/1471-2407-5-138)
Supplement: Additional File 1 — The distribution of the numbers of metastases. [file 1471-2407-5-138-S1.pdf]

Additional file 1

The distribution of the numbers of metastases

A model for the frequency distribution of the number of hematogenous organ metastases has previously been validated, where the numbers of organ metastases is assumed to be affected by Poisson counting statistics and by stochastic variations in regional organ blood flow [29]. The assumptions inherent to this model will be summarized here, but the reader is referred back to the initial article for additional details.

Physiologists have long recognized that the amount of blood flow within a tissue, as measured by the number of entrapped radiolabelled microspheres, obeys a power function relationship with respect to the mass of the tissue samples [33]. This empirical relationship can be based upon a stochastic model for blood flow [28], using the theory of exponential dispersion models—a class of statistical models used to describe a range of non-normal data [54]. This blood flow model was based upon a compound Poisson gamma (PG) distribution, which represented the sum of a Poisson number of independent gamma distributions. The number of potential sites for microsphere entrapment was assumed distributed throughout tissue in accordance with a Poisson distribution, whereas the blood flow to these sites was assumed to obey a gamma distribution. The blood flow was further assumed to obey a PG distribution regardless of the size of the sample, a restriction that implied a power function relationship between the variance and mean, in keeping with the empirical observations.

The number of hematogenous metastases can be represented by a discrete random variable that depends proportionately on the continuous variable, blood flow, and thus reflects the empirically observed power function relationship seen with blood flow. We have then a relationship between the mean number of metastases *E(Z)* and its variance *var(Z)* such that,

$$\mathrm{var}(Z) = \lambda^{1/(1-\alpha)} E(Z)^p + E(Z),$$

where the power function exponent $p = (\alpha - 2)/(\alpha - 1)$. The related distribution can be described by a Poisson negative binomial (PNB) distribution [29]. This PNB distribution has the probability generating function (PGF),

$$G(x) = \exp\left[\lambda \frac{\alpha - 1}{\alpha}\left(\frac{\theta}{\alpha - 1}\right)^{\alpha}\left\{\left(1 - \frac{1}{\theta} + \frac{x}{\theta}\right)^{\alpha} - 1\right\}\right].$$

The parameters α, λ, and θ are constants used to characterize the distribution and x is an abstract variable used to construct the PGF. (Parenthetically, it should be mentioned that this model does not account for the possibility of metastasis of metastases.)

The parameters within the PGF can be related to observations drawn from repeated animal experiments. Provided the mean and variance of the numbers of metastases and the corresponding power law exponent p can be assessed, one can estimate α, λ, and θ. We then have the relationships

$$\alpha = (p - 2)/(p - 1),$$

$$\lambda = (\mathrm{var}(Z) - E(Z))^{\alpha - 1} / E(Z)^{\alpha - 2},$$

and

$$\theta = (\alpha - 1)(E(Z)/\lambda)^{1/(\alpha-1)}.$$

The parameters used within the present simulation were thus derived from observations derived from controlled murine experiments by these means.

The discrete probability density p_n for n number of metastases can be obtained from the PGF through its Taylor series expansion, $p_n = G^{(n)}(0)/n!$. This was done here numerically using a symbolic manipulating program, MAPLE 8 (Waterloo Maple Inc., Waterloo, Ontario, Canada), with the parameter choices $\alpha = -1.03$, $\lambda = 0.2$, and $\theta = -0.331$. These parameters were chosen to so that the mean and variance of the distribution would fit within the observed range from experimental metastasis assays of B16 F10 murine melanoma, which had been injected under identical conditions into age-matched syngeneic mice [25].

The numerical values for the probability density were summed to provide a cumulative distribution function (CDF). This theoretical CDF was inverted numerically against simulated values drawn from a uniform distribution of pseudorandom numbers ranging from 0 to 1 [55]. By this means, a simulated set of values could be obtained by the Monte Carlo technique to represent the numbers of metastases expected by chance, granted that each exfoliated tumor clonogen had the same propensity to form a metastasis and that the host organ provided the same conditions for metastatic colony formation.

The distribution of the sizes of metastases

A model for the size distribution of human hematogenous metastases has also been derived and validated, based on the hypothesis of normally distributed growth times for exponentially growing metastatic colonies within an individual with cancer [31]. The theoretical reasons for postulating normally distributed growth times will be summarized from this article here. With the transformation event, a primary cancer will presumably originate from a single cell. Over time the resultant progeny cells will reproduce; some cells may die; other cells may eventually metastasize. The formation of a metastasis requires the completion of a number of additional steps: invasion, exfoliation, transfer through the vascular tree, entrapment within an end organ, adherence to the capillary endothelium of the end organ, invasion through this endothelium, angiogenesis, and so on [35, 36]. In any individual the transformation event and the time that the resultant metastases are assessed can be considered to be two fixed time points. One may argue on the basis of the Central Limit Theorem and the presumed summed times required for the multiple sequential steps, that the time between the transformation event to the inception of each metastasis should be normally distributed. Consequently, the time available for the growth of the metastasis up to its assessment would also be normally distributed.

If one assumes that the growth of the metastases within any individual occurs exponentially and at the same rate, the resultant distribution for their sizes would approximate a lognormal form. To conduct this simulation here a fully stochastic model for metastatic colony formation was further specified, where the individual metastases expanded in numbers according to a stochastic pure birth process [34]. Each metastatic

colony was assumed to originate from a single clonogen, and so the probability that a colony would leave i number of descendents would then be

$$P_i(t) = \begin{cases} 0 & \text{if } i = 0 \\ e^{-\lambda t}(1 - e^{-\lambda t})^{i-1} & \text{if } i \geq 1 \end{cases}.$$

Here λ represents the birth rate and t is the time available for colony growth. If we set $\lambda = \ln(2)$ then the growth times would be measured in units of cell doublings.

The probability that a lineage leaves i descendents, given that the time available for growth is normally distributed, is then

$$N_i = \int_0^\infty P_i(t) \frac{e^{-(t-m)^2/2\sigma^2}}{\sqrt{2\pi}\sigma} dt \Big/ \int_0^\infty \frac{e^{-(t-m)^2/2\sigma^2}}{\sqrt{2\pi}\sigma} dt \, .$$

Here m is the mean time available for colony growth, and σ is the respective standard deviation. In the simulations herein the mean time was arbitrarily set at $m = 16$ doublings with a standard deviation of $\sigma = 1$ doubling. The resultant probability values were used to construct a theoretical CDF, which then was utilized in accordance with the Monte Carlo method [55], as outlined above, to simulate the number of cells per metastasis.

In this simulation the growth rates of the individual metastases were assumed equal and constant. There is abundant experimental and clinical evidence that metastases can propagate with different rates within an individual. Although this assumption was made for the purposes of simplification, if it were to be relaxed the result would be a greater spurious variability in the sizes of metastases within an individual.
